# Head and Tail Entity Fusion Model in Medical Knowledge Graph Construction: Case Study for Pituitary Adenoma

**DOI:** 10.2196/28218

**Published:** 2021-07-22

**Authors:** An Fang, Pei Lou, Jiahui Hu, Wanqing Zhao, Ming Feng, Huiling Ren, Xianlai Chen

**Affiliations:** 1 Life Science College Central South University Changsha China; 2 Institute of Medical Information Chinese Academy of Medical Sciences Beijing China; 3 Peking Union Medical College Hospital Chinese Academy of Medical Sciences Peking Union Medical College Beijing China; 4 Big Data Institute Central South University Changsha China; 5 National Engineering Lab for Medical Big Data Application Technology Central South University Changsha China

**Keywords:** knowledge graph, pituitary adenoma, entity fusion, similarity calculation

## Abstract

**Background:**

Pituitary adenoma is one of the most common central nervous system tumors. The diagnosis and treatment of pituitary adenoma remain very difficult. Misdiagnosis and recurrence often occur, and experienced neurosurgeons are in serious shortage. A knowledge graph can help interns quickly understand the medical knowledge related to pituitary tumor.

**Objective:**

The aim of this study was to develop a data fusion method suitable for medical data using data of pituitary adenomas integrated from different sources. The overall goal was to construct a knowledge graph for pituitary adenoma (KGPA) to be used for knowledge discovery.

**Methods:**

A complete framework suitable for the construction of a medical knowledge graph was developed, which was used to build the KGPA. The schema of the KGPA was manually constructed. Information of pituitary adenoma was automatically extracted from Chinese electronic medical records (CEMRs) and medical websites through a conditional random field model and newly designed web wrappers. An entity fusion method is proposed based on the head-and-tail entity fusion model to fuse the data from heterogeneous sources.

**Results:**

Data were extracted from 300 CEMRs of pituitary adenoma and 4 health portals. Entity fusion was carried out using the proposed data fusion model. The F1 scores of the head and tail entity fusions were 97.32% and 98.57%, respectively. Triples from the constructed KGPA were selected for evaluation, demonstrating 95.4% accuracy.

**Conclusions:**

This paper introduces an approach to fuse triples extracted from heterogeneous data sources, which can be used to build a knowledge graph. The evaluation results showed that the data in the KGPA are of high quality. The constructed KGPA can help physicians in clinical practice.

## Introduction

Pituitary adenoma is one of the most common central nervous system tumors. Most of the benign adenomas are characterized by swelling growth, which can be cured by surgery or medicine [1]. However, a small number of pituitary adenomas are not sensitive to surgery, radiotherapy, and drug therapy, and metastasis will lead to pituitary adenocarcinoma [2]. At present, there are difficulties in the diagnosis and treatment of pituitary adenoma [3]. In some cases, pituitary adenocarcinoma can even be life-threatening [4] and the prognosis is extremely poor. Therefore, pituitary adenoma has become a hot topic in life science research, and an open knowledgebase of pituitary adenoma is needed.

A knowledge graph is a general framework for formal description of knowledge, which can describe knowledge in the form of triples as a “head entity-relation-tail entity,” one of the most popular knowledge representation methods currently adopted [5]. Well-known open-domain knowledge graphs include Freebase, DBpedia, YAGO, and NELL, among others [6]. Knowledge graphs are also widely used in the medical field. Gong et al [7] proposed a method to build a diabetes knowledgebase by mining the web; they extracted knowledge from the semistructured content of the vertical portal and then mapped the information onto a unified knowledge graph. Ernst et al [8] constructed a biomedical science knowledge graph in which they extracted data using distant supervision methods and used logical reasoning for consistency checks. Rotmensch et al [9] designed an automatic extraction framework to directly extract diseases and symptoms from electronic medical records (EMRs), and automatically constructed a knowledge graph.

Data fusion is an important step of the integration of heterogeneous data in the construction of knowledge graphs. Entity fusion includes methods based on character similarity, clustering, deep learning, and others. Zhang et al [10] proposed a novel multisource medical data integration and mining solution for better health care services, which can search for similar medical records in a time-efficient and privacy-preserving manner. Wang et al [11] extracted different semantic words using multimodal trees and performed multigranularity feature fusion on the data. Li et al [12] proposed a novel fusion-embedding learning model, G2SKGE, which aims to learn the subgraph structure information of the entity in a knowledge graph. Li et al [13] proposed an approach to build a knowledge graph for hepatocellular carcinoma, and applied a biomedical information extraction system to filter and fuse the data.

In this study, we extracted data from patient EMRs and medical websites, fused the entities using our proposed head-and-tail entity fusion model, and constructed a medical knowledge graph for pituitary adenoma (KGPA). The main contributions of this study are as follows. First, there is currently no Chinese knowledgebase for pituitary adenoma. Therefore, this study presents the complete process of knowledge graph construction, which was used to construct the KGPA. Second, to integrate the data extracted from different sources, we propose a fusion method suitable for medical data that was used in the process of KGPA construction. The method includes two steps: tail entity fusion and head entity fusion. Finally, knowledge of pituitary adenoma, such as the typical symptoms of different pituitary adenoma–related diseases, can be clearly revealed by searching the KGPA. According to doctors’ feedback on use of the KGPA, the content displayed in the KGPA was considered to be consistent with the actual clinical situation.

## Methods

### Overview

**Figure 1:**
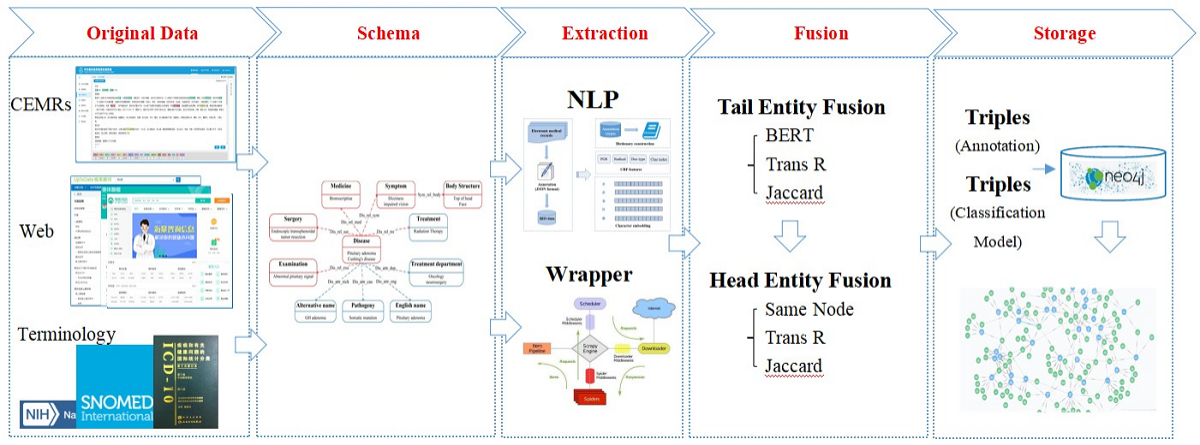
Process for construction of the knowledge graph for pituitary adenoma. CEMR: Chinese electronic medical record; NLP: natural language processing; BERT: bidirectional encoder representations from transformer.

According to the characteristics of pituitary adenoma diseases combined with the characteristics of Chinese electronic medical records (CEMRs) and Chinese health websites, we designed the construction framework of the KGPA, as shown in Figure 1, which includes 5 steps: raw data collection, schema design, data extraction, data fusion, and data storage and visualization. Each step is introduced in detail below, with emphasis on the proposed data fusion model.

### Data Schema

The knowledge graph includes a data layer and a schema layer [14]. Entities, relations, and attributes in the data layer are regulated and restricted by the schema. The schema was based on several open-access authoritative terminologies and ontologies, including the UMLS Semantic Network [15], the concept definitions in SNOMED-CT [16], and the International Statistical Classification of Diseases and Related Health Problems (ICD-10). In addition, the natural language processing datasets defined by the Informatics for Integrating Biology & the Bedside [17] and CEMRs Entity and Relations Annotation Specifications defined by Harbin Institute of Technology [18] were also referenced for this task. With the help of clinical experts, a combination of top-down and bottom-up approaches was used to construct the KGPA schema.

**Figure 2:**
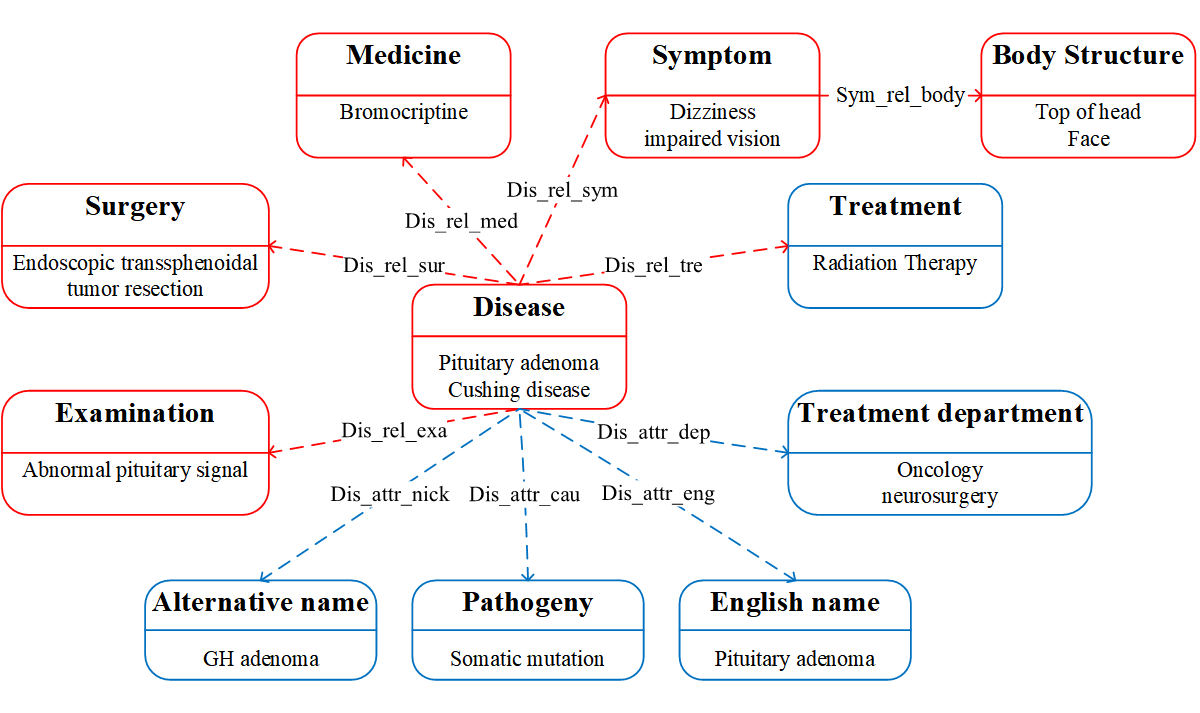
Schema of the knowledge graph for pituitary adenoma (KGPA). Concepts extracted from Chinese electronic medical records are in red. Concepts extracted from health websites are in blue. GH: growth hormone.

In our previous study of CEMRs data extraction, we found that the medical diagnosis and treatment activities could be summarized based on symptoms (symptom) and abnormal results (examination) [19]. The doctor will give a comprehensive diagnosis conclusion (disease) and corresponding treatment measures (surgery, medicine). Therefore, the mentioned entities and the relations between them were abstracted for design of the schema. The CEMRs are detailed but contain a limited number of concepts; therefore, we extracted data from medical websites to expand the concepts. Through analyzing the data types of the websites, six types of concepts were added to the schema: pathogeny, treatment, examination, treatment department, English name, and alternative name. The most frequently used disease term in websites was selected as the concept of the disease, and then treatment and examination were defined as related entities. Pathogeny, treatment department, English name, and alternative name were defined as the attributes of the disease. Attributes can be used to describe the internal characteristics of the disease entities; the more attributes there are, the more complete the information of the entity will be [20]. The KGPA schema is shown in Figure 2.

### Data Extraction

#### Process

In the process of data extraction, entities and relations were first extracted from unstructured information in CEMRs. For website data, specific HTML wrappers were constructed to directly extract the triples (eg, Cushing syndrome, Symptom, Lethargy). The details are described below.

#### EMR Data Extraction

CEMRs include information on admission, discharge summary, disease course, and a medical record summary, among other details. Since the history of present illness (HPI) in the admission record contains a large amount of detailed patient symptoms and preliminary examination information, the HPI was selected as the main data source in our study.

**Figure 3:**
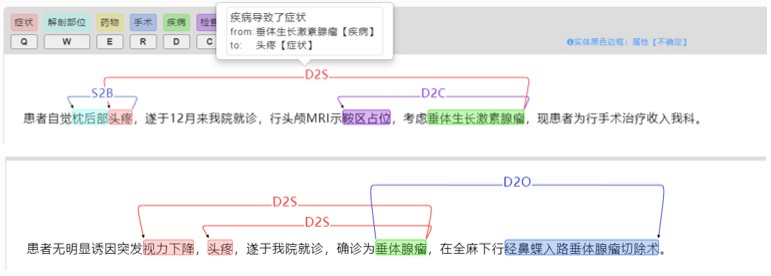
Medical text annotation using the Chinese Clinical Natural Language Processing System (CCNLP) system.

The Chinese Clinical Natural Language Processing System (CCNLP) [21] developed by our team was used to annotate entities and relations in CEMRs, as shown in Figure 3. The CCNLP allows user to customize the entities and relations. According to the definition of the schema, we defined 6 types of entities and 5 types of relations in the CCNLP. Two clinicians were invited to perform annotation. The conditional random field model is embedded in the system, which can train the annotated corpus and assist in annotation. The results of the two annotators were evaluated by the consistency evaluation function of the CCNLP [22].

#### Web Data Extraction

The web data were mainly collected from medical websites and high-quality encyclopedia websites. The extracted disease entities in the CEMRs were used as search terms on the medical websites. Since single medical website retrieval is not comprehensive, four websites with higher data quality were used: xywy [23], UpToDate [24], Baidu Encyclopedia [25], and chunyuyisheng [26]. All of these websites provide HTML pages of diseases, symptoms, treatments, and other relevant details. This enabled obtaining sufficient medical knowledge to construct the knowledge graph.

**Figure 4:**
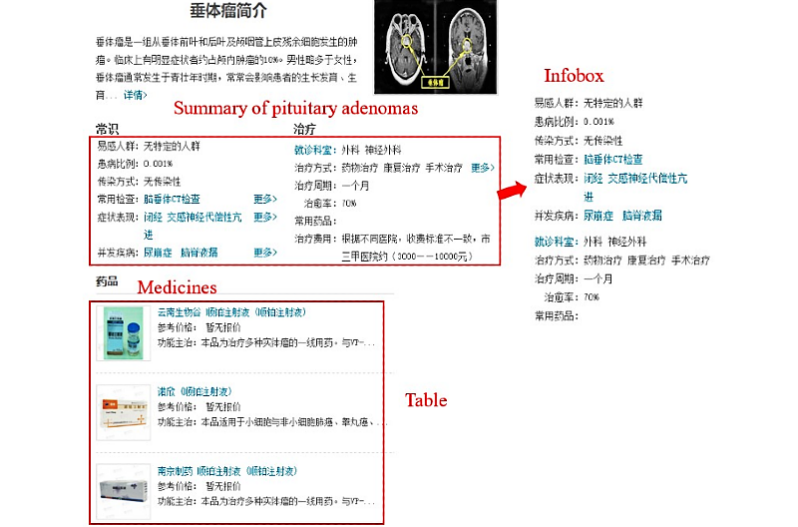
Web page structural analysis for knowledge extraction.

Since the websites shared similar structures, xywy was selected as an example to illustrate the details of pages and its structures used for data extraction. As shown in Figure 4, the information in “Infobox” can be directly extracted and stored as triples. The “Medicines” data in the website are stored in a tabular format. We extracted the title and first lines of the tables, which were combined as triples. Different wrappers were designed to extract information from different web pages.

### Data Fusion

#### Framework

**Figure 5:**
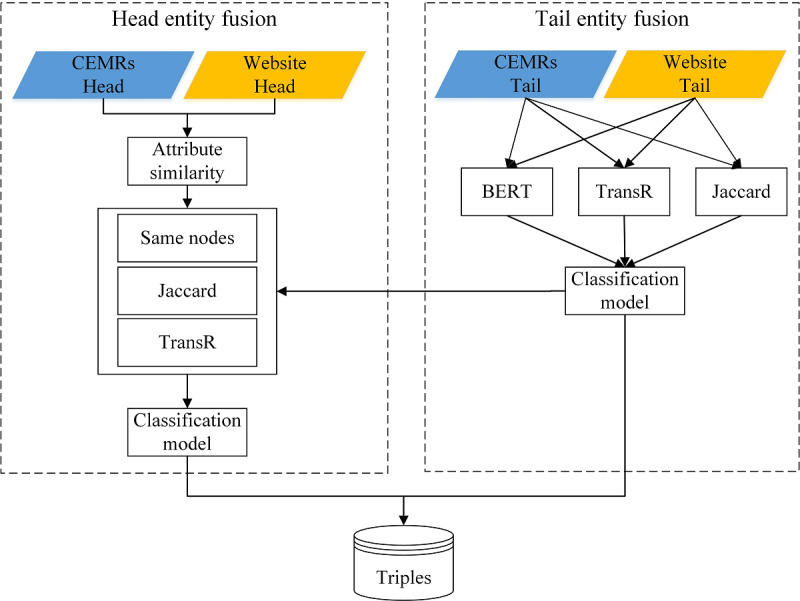
Data fusion framework. CEMR: Chinese electronic medical record; BERT: bidirectional encoder representations from transformer.

Triples from different sources may have complements, redundancies, or even conflicts among each other. To ensure accuracy of the data in the knowledge graph, a data fusion method was proposed as shown in Figure 5. The data were fused by calculating the similarity of head entities and tail entities. The purpose of similarity calculation is to find the optimal alignment between the website entities and CEMR entities. The fusion methods were carried out in two steps. First, the similarity of tail entities (symptoms and examinations contained in both data sources) were calculated based on bidirectional encoder representations from transformer (BERT), the TransR model, and the Jaccard coefficient. Tail entity fusion enabled obtaining a more consistent entity expression. Second, the structural information of the graph was used to merge the head entities (diseases) through the TransR model, Jaccard coefficient, and the count of same nodes.

#### Tail Entity Fusion Model

##### Features

In the entity fusion task, there are only two types of training results (positive and negative); therefore, this can be converted into a binary classification problem. In the tail entity fusion experiment, three different features were constructed as model inputs: semantic similarity, TransR similarity, and Jaccard similarity.

##### Semantic Similarity Calculation Based on BERT

**Figure 6:**
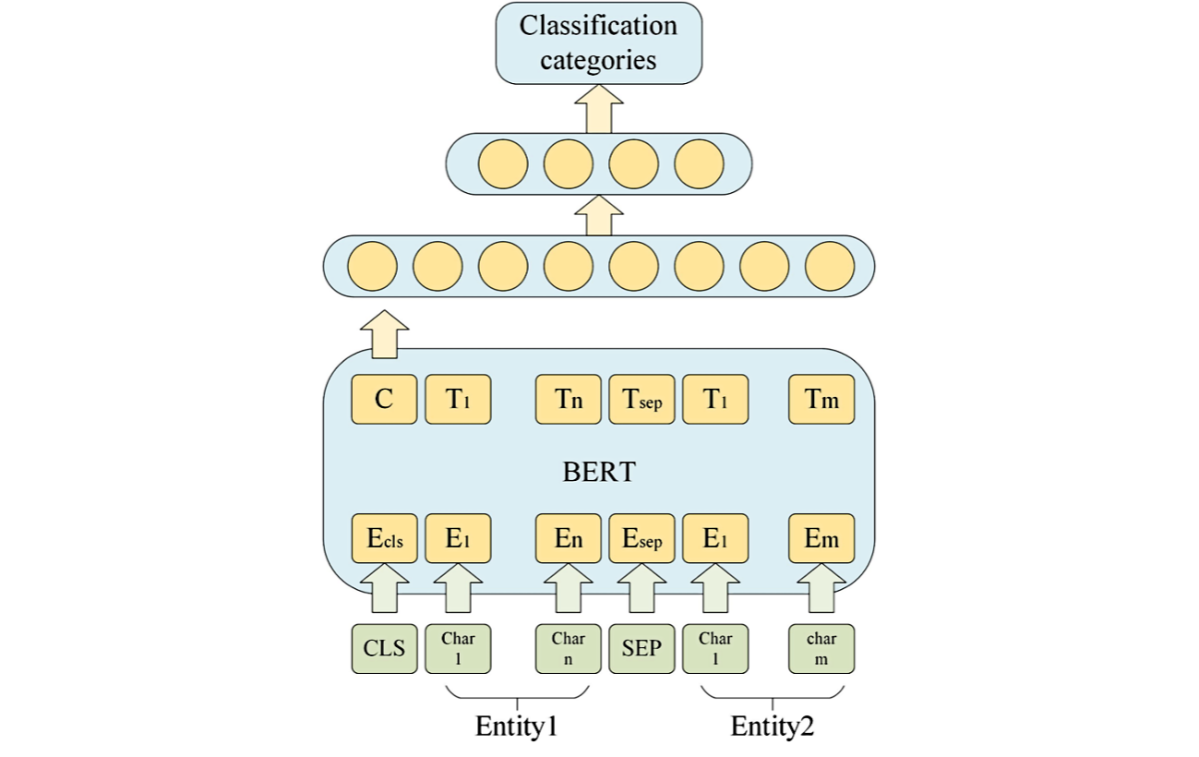
Semantic similarity calculation model based on bidirectional encoder representations from transformer (BERT).

A semantic model is widely used in the similarity calculation of textual data. In this study, the semantic classification model was trained with labeled data. BERT-Base, Chinese [27] was used to construct the embedding of the tail entities in CEMRs and website data, as shown in Figure 6. Tail entities can be regarded as short sentences, and the matching problem of entity pairs can be modeled as a classification task. The first output vector of the coding layer “C” is taken as the semantic representation of the entity pair. “[CLS]” represents the beginning of a sentence and “[SEP]” separates the two sentences. “E” represents the word embedding of the input character and “T” represents the contextual representation of the input character. The semantic categories are then calculated using two full connection layers: full connection layer 1 uses a tanh activation function and full connection layer 2 normalizes the probability of each class with the softmax function.

##### Knowledge Representation Learning

Knowledge representation learning methods do not rely on textual information but rather obtain the depth characteristics of the data by mapping the entities to low-dimensional space vectors. A total of 4684 pituitary adenoma triples were used to test the data representation ability of the Trans models [28]. We evaluated the performance of the models using hits@10 (ie, the proportion of correctly aligned entities ranked in the top 10 predictions); a higher hits@10 value indicates better performance. The evaluation results were 0.27 for TransE, 0.37 for TransH, and 0.39 for TransR. Therefore, TransR was selected for knowledge representation learning. The extracted triples were used as positive examples (head [*h*], relation [*r*]*,* tail [*t*]). For each positive triple, we randomly replaced its head entity (*h’, r, t*) or tail entity (*h, r, t’*) to generate a negative triple. A mapping matrix *M_r_* was used to describe the relational space of relation *r*. Using the gradient descent method to update the parameters, we obtained the vector of the tail entities *trans_vec*. The cosine similarity cos was used to calculate the tail entity similarity of the two data sources, as shown in Equation 1:

Simteal_trans(m_i_,n_i_)=argmax(cos[trans_vec_mi_],cos[trans_vec_ni_])

**(1)**

##### Jaccard Coefficient

The Jaccard coefficient was selected as the third feature of tail entity fusion. The Jaccard coefficient refers to the ratio of the number of intersection elements to the union elements in two sets; the higher the Jaccard value, the higher the similarity. We assigned each tail entity in the CEMRs and websites to sets *t_1_* and *t_2_*, respectively. The Jaccard coefficient represents the ratio of the same number of Chinese characters in the two words to the total number of characters, as shown in Equation 2:

Jaccard(t_1_,t_2_)=|t_1_∩t_2_|/|t_1_|+t_2_|–|t_1_∩t_2_|

**(2)**

#### Head Entity Fusion Model

##### Features

When merging head entities (diseases), the similarity of the two attributes and their structures were mainly considered. That is, if two head entities are the same, their neighboring entities should also be similar.

##### Attribute Similarity

Entity alignment can be performed using the alternative name attribute or the English name attribute of the disease. If the head entities in the two data sources have the same alternative name or English name, the two entities can be considered the same. For example, “垂体生长激素腺瘤” (growth hormone–secreting pituitary adenoma) has alternative names of “pituitary growth hormone secreting adenoma” and “GH adenoma.” Therefore, we can align “pituitary growth hormone secreting adenoma” and “GH adenoma” to “growth hormone–secreting pituitary adenoma.”

##### Structural Similarity Fusion Model

When the head entities cannot be aligned by the attribute, we propose using the structural similarity model to fuse entities. Three different features were chosen as the classifier model’s inputs: the number of identical tail nodes, Jaccard similarity, and TransR similarity, as shown in Equation 3.

The head entity and the tail entity have a 1-N relationship. Taking two disease sets 
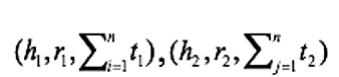
 from two data sources as an example, 
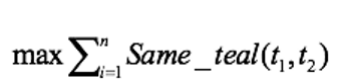
 represents the number of identical tail nodes in different sets and 
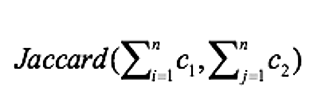
 represents the ratio of the same number of characters to the total number of characters of two sets. The order of words in the set is not considered. For the attribute similarity, the vector representation of entities was trained using the TransR model, whereas in this case, we calculated the vector of the head entity using the TransR model.



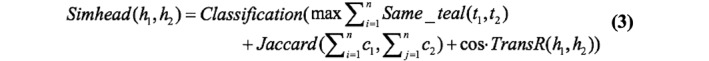



After the head entities of two heterogeneous data sources were fused, the triples containing all of the disease information were obtained. Finally, to standardize the disease names in the knowledge graph, we mapped them to the ICD codes.

## Results

### Data Extraction

Three hundred clinical medical records and 4 portal websites were selected as data sources to construct the KGPA. Although these are all Chinese resources, our proposed approach is not dependent on a particular language and can be applied to data resources in other language in the same way. The data in CEMRs were annotated by two doctors using the CCNLP system [21]. With the consistency test function of the system, the consistency of the annotations reached 95.2%. Website data were extracted according to the wrapper defined in this study. Table 1 shows the number of all entities extracted from the two types of data sources. The concepts are abundant in websites, whereas the CEMRs included more symptom entities, which can help to expand more data types for the KGPA. The “Prefusion” column of Table 1 shows the number of all relations extracted from the two types of data sources.

**Table 1:** Number of relations before and after data fusion.

Relation	Head entity	Tail entity	Prefusion	After fusion
Diseases_rel_Symptom	disease	symptom	3154	1940
Diseases_rel_Surgery	disease	surgery	55	45
Diseases_rel_Medicines	disease	medicine	245	182
Diseases_rel_Examination	disease	examination	437	274
Symptoms_rel_Body structure	symptom	body	396	281
Diseases_rel_Treatment	disease	treatment	110	109
Diseases_attr_Pathogeny	disease	pathogeny	122	104
Diseases_attr_Department	disease	department	71	44
Diseases_attr_English name	disease	English name	71	42
Diseases_attr_Alternative name	disease	alternative name	23	20

### Data Fusion

Two hundred medical records were randomly selected for the fusion experiment. The ratio of the training set and test set was 8:2. The experiment was trained under Windows 10, and the model based on the TensorFlow framework was used.

The proposed tail entity fusion model was used to perform entity fusion for symptoms and examinations. Before the fusion began, different entities with the same conceptual semantics extracted from different websites were merged to reduce duplication and computation. A vector representation of 768 dimensions was constructed through the Chinese BERT model, and then the similarity results were obtained by full connection layers. A 50-dimensional vector was obtained by the TransR model and the cosine similarity was used to calculate the entity pair similarity values. The Jaccard coefficient was used as a numerical feature. These three results were taken as features into the classification model. Three different classification models were adopted for training: logistic regression, decision tree, and neural network. The results are shown in Table 2. The neural network showed the best performance.

Subsequently, the triples completed by the tail entity fusion model were used for the head entity fusion experiment. A total of 65 head entities were fused between CEMRs and websites. Among them, 17 entities could be directly mapped by disease name, 6 entities could be fused by attribute (eg, growth hormone–secreting pituitary adenoma, pituitary microadenoma, Cushing syndrome, hypothyroidism), and 42 head entities were fused based on the proposed structural similarity fusion model. The three classification models above were used for training. As shown in Table 2, the decision trees performed better when fusing head entities because the data inputs to the model were smaller than the fusing tail entities. With the increase of data volume, the advantages of the neural network were reflected in the fusion of tail entities.

Additionally, we divided the features into four variants for an ablation study. We selected logistic regression as the classification model to explore the contribution of different features to the model, and these results are also shown in Table 2. These three features had nearly the same contributions to the model in the head entity fusion. For a specific disease knowledge graph, the Jaccard similarity feature played a major role in the tail entity ablation experiment, and the features based on BERT and TransR simply contributed by fine-tuning the model.

Table 3 shows that our proposed model has higher accuracy than previous models. Compared with previous models, we divided the entities into head entities and tail entities and fused them according to different characteristics. Different concepts were considered separately in the step-by-step fusion process, which improved the precision of the fusion.

**Table 2:** Head and tail fusion model performance.

Fusion model					Precision (%)			Recall (%)			F-score (%)
**Head entity fusion**											
	**Linear regression models**										
		Ja^a^+TransR	83.37			84.06			83.71		
		Sa^b^+TransR	83.37			84.55			83.95		
		Ja+Sa	83.85			84.55			84.19		
		Ja+Sa+TransR	83.92			84.61			84.26		
	Neural network			97.29			97.03			97.16	
	Decision tree			97.47			97.18			97.32	
**Tail entity fusion**											
	**Linear regression models**										
		BERT^c^+TransR	61.73			61.74			61.73		
		Ja+BERT	95.76			95.83			95.79		
		Ja+TransR	95.89			95.93			95.90		
		Ja+BERT+TransR	95.92			95.94			95.93		
	Neural network			98.43			98.72			98.57	
	Decision tree			98.18			98.05			98.11	

^a^Ja : Jaccard similarity.

^b^Sa: identical tail nodes in different sets: 
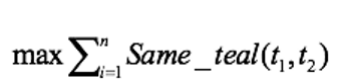
.

^c^BERT: bidirectional encoder representations from transformer.

**Table 3:** Model comparison.

Model	Research field	Method	F1-score
Ruan et al [29]	Symptom	Align entities according to the string similarities of the entity names and attribute values	—^a^
Yang et al [30]	Disease, medicine	Align entities according to the entity’s attribute types (attr_bool_, attr_numeric_, attr_string_, attr_time_)	0.60
Sun et al [31]	Disease, medicine, symptom	Character similarity of entity pairs and degree centrality of entities in the graph	0.76
Liu et al [32]	Disease, medicine, examination	Semantic classification model based on pretrained BERT^b^	0.83
Our model	Symptom, examination, disease	Multifeature learning based on head-and-tail entities	0.97

^a^Not provided.

^b^BERT: bidirectional encoder representations from transformer.

The triples obtained after data fusion were stored and visualized in Neo4j [33]. The KGPA contained 1789 entities and 3041 pairs of relations of 73 pituitary adenoma–related diseases. For a knowledge graph, accuracy is of great importance. However, there is currently no gold standard for pituitary adenoma knowledge graph validation. To evaluate the quality of the knowledge graph, the accuracy of triples was used as an indicator. Three hundred triples were randomly sampled and each triple was manually evaluated by two physicians; the accuracy reached 95.4%.

## Discussion

### Principal Findings

A knowledge graph was constructed by mining CEMRs and web resources. In the process of KGPA construction, to solve the problem of knowledge duplication between heterogeneous data sources, we proposed a head-and-tail entity fusion model. The model showed good performance on the fusion of medical data.

**Figure 7:**
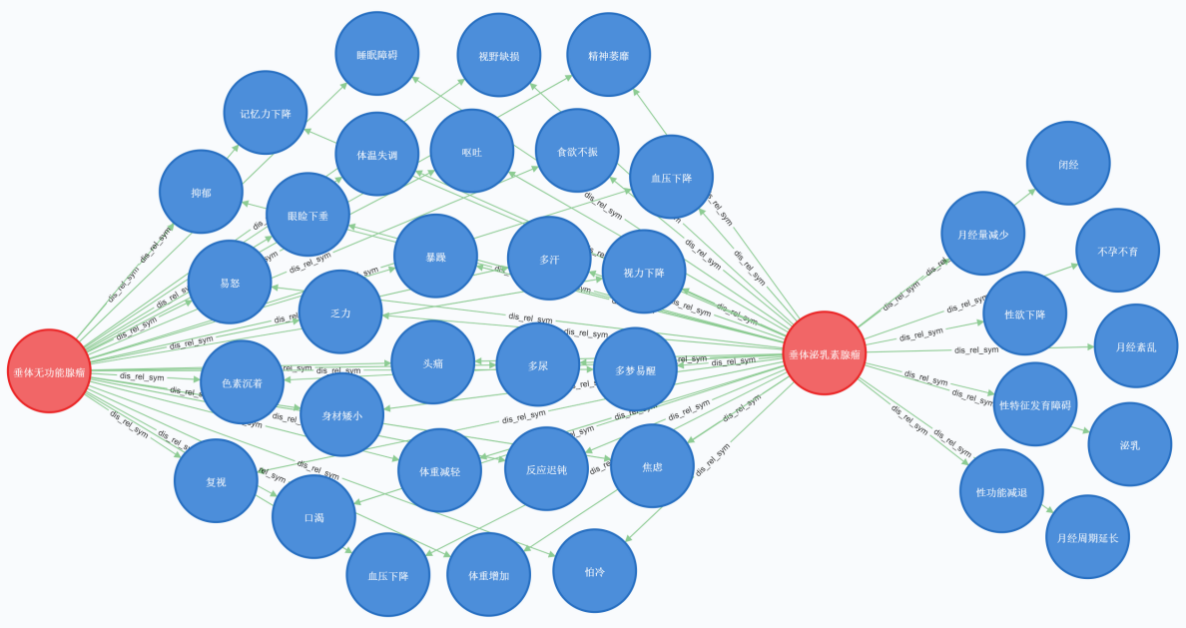
Differences of typical symptoms between “prolactin-secreting pituitary adenomas” and “nonfunctioning pituitary adenoma” in the knowledge graph for pituitary adenoma.

The KGPA was proven to be effective when displaying the typical symptoms of pituitary adenoma–related diseases. For example, the query for symptoms of disease “prolactin (PRL)-secreting pituitary adenomas” differed from the query for the disease “nonfunctioning pituitary adenoma” using the following query in Cypher: “MATCH (p:dis{disease: 垂体泌乳素腺瘤})-[:dis_rel_sym]->(n), (m)<-[:dis_rel_sym] -(q:dis{disease:垂体无功能腺瘤}), WHERE (m)<>(n), RETURN p,n,q.” As shown in Figure 7, the entities in the middle of the graph are symptoms of both diseases and the entities on the right are typical symptoms unique to the disease “PRL-secreting pituitary adenomas.”

Searching for the KGPA by Cypher, we found that most pituitary adenoma–related diseases have the following basic symptoms: headache, vision problems, fatigue, slow reaction, mood problems, changes in height and weight, changes in appetite, and changes in sleep. Nonfunctioning pituitary adenoma has all of these basic symptoms listed above. In addition to the basic symptoms, pituitary thyroid-stimulating hormone adenoma is also associated with symptoms of goiter, palpitation, and exophthalmos. The typical symptoms of PRL-secreting pituitary adenomas are associated with the reproductive system, decreased libido, and menstrual changes in women. The typical symptoms of pituitary growth hormone adenoma are altered facial features, enlarged hands and feet, snoring, and metabolic disorders. Cushing syndrome is characterized by obesity, altered skin color, increased hair, and edema. Based on clinicians’ feedback on the use of the KGPA, the knowledge in the KGPA was consistent with the actual clinical situation. The KGPA will be useful for clinical interns in diagnosis and treatment, and may also be helpful for medical students to quickly master knowledge of pituitary adenoma–related diseases.

### Limitations

The KGPA was constructed by integrating CEMRs and web data related to pituitary adenoma. However, since we only focused on pituitary tumors, the data volume was relatively small. In the next step, we plan to try to extend the method proposed in this study to the entire neurosurgery field or even larger fields and apply the knowledge graph to clinical practice.

### Conclusion

This study shows that entities and relations extracted from heterogeneous data sources such as CEMRs and health websites can be used to construct a knowledge graph after entity fusion. The head-and-tail entity fusion model proposed in this paper achieved 97% in accuracy, which is higher than that reported for previous models. The KGPA constructed in this study can be used to discover the knowledge hidden in the source text, such as typical symptoms unique to the disease “PRL-secreting pituitary adenomas.” Based on clinicians’ feedback, the knowledge in the KGPA was consistent with the actual clinical situation. The knowledge graph constructed will be useful and helpful for patients, medical students, and interns to assist in obtaining information for symptoms, diagnosis, treatment, and disease pathogenesis.

## Abbreviations

BERT: bidirectional encoder representations from transformer
CCNLP: Chinese Clinical Natural Language Processing System.
CEMR: Chinese electronic medical record
EMR: electronic medical record
HPI: history of present illness
ICD: International Classification of Diseases
KGPA: knowledge graph for pituitary adenoma
PRL: prolactin
